# Tradeoffs Among Predator Control, Moose Harvests, and Trophy Antlers: Principles Pertinent to Managing Alaska’s Wildlife

**DOI:** 10.3390/ani16030472

**Published:** 2026-02-03

**Authors:** R. Terry Bowyer, Sterling D. Miller, David K. Person

**Affiliations:** 1 Institute of Arctic Biology, University of Alaska Fairbanks, Fairbanks, AK 99775, USA; 2 Alaska Department of Fish and Game, Anchorage, AK 99519, USA; sterlingmil@gmail.com; 3 Alaska Department of Fish and Game, Juneau, AK 99802, USA; davekp11@gmail.com

**Keywords:** Alaska, black bear, grizzly bear, intensive management, moose, predator control, tourism, trophy hunting, wolves

## Abstract

The State of Alaska, USA, has a long and controversial history of controlling predators to enhance ungulate populations, including moose (*Alces alces*). Moose management is complicated by a dual system in which the Federal and State governments prioritize harvesting moose for human consumption over other considerations, such as trophy or sport hunting, but have conflicting regulations regarding who is eligible to harvest moose. Wildlife management for the State is overseen by the Alaska Board of Game (BOG), with advice from the Alaska Department of Fish and Game (ADFG). In accordance with its Intensive Management Policy, the BOG establishes regulations promoting the harvest of moose and other ungulates for human consumption. This typically occurs by controlling bears (*Ursus americanus* and *U. arctos*) and gray wolves (*Canis lupus*) in anticipation of increasing ungulate harvests, often without adequate information on the status and ecology of predator or ungulate populations. We provide a narrative and integrative review of moose population dynamics to help resolve those issues. We argue that the current management of moose and their predators in Alaska does not encompass a full range of management options and fails to consider or implement important aspects of their population dynamics. Predators maintain some moose populations at a low density, reducing the harvest of moose but promoting large-antlered individuals, which are of value to Alaska’s professional guide and tourism industries. Using modern models of population dynamics of moose and other ungulates, we argue that if the proximity of the moose population to *K* (the ecological carrying capacity) is known, management strategies that increase the human harvest of moose and also promote large-antlered trophies are not mutually exclusive. We list life history and population characteristics to help determine the nutritional status of moose populations in relation to *K*, thereby guiding wise management of that valuable resource. We also recommend an adaptive management approach to assessing the effects of such activities. We caution, however, that to wisely manage these important wildlife resources, more information on the dynamics of moose and their predators is necessary. A system that embraces more biology and fewer politics would provide greater opportunities to employ the best science in the management of moose and their predators.

## Simple Summary

Alaska’s Intensive Management law prioritizes the harvest of moose and other ungulates for human consumption over other purposes. This policy, however, does not consider density-dependent processes, whereby the productivity of moose is a result of population size in relation to the carrying capacity of the habitat. Wolves and bears may reduce moose to low numbers, but predator control efforts augmenting moose populations have not increased the harvest of moose and can be controversial. Mostly male moose are harvested, which has little effect on the productivity of populations and, hence, the amount of meat obtained. Moose at low densities are on a high nutritional plane because of reduced competition, resulting in males with trophy-sized antlers, which is of value to hunters and supports tourism. Once moose achieve moderate densities, larger numbers of moose (and more meat) would result from harvesting females; males would also possess large antlers. Moose populations that reached a high density because of predator control would be nutritionally stressed, females would exhibit low productivity, and males would possess smaller antlers. We proposed an adaptive management approach that adjusts management to reflect the population characteristics of moose, thereby incorporating the best available science to manage moose and their predators.

## 1. Introduction

Our purpose is to evaluate the current management of moose (*Alces alces gigas*) in Alaska, USA, in light of the most current scientific understanding of ungulate population dynamics in the presence of predation. To do so, we provide a narrative and integrative review of moose population dynamics and predator management in Alaska. Extensive literature reviews for moose [1,2] and ungulate population dynamics [3,4,5,6,7] are available. We relied heavily on these publications and their citations to help identify and integrate the important concepts discussed. Indeed, misunderstanding or misapplying concepts related to managing predators and harvesting moose can undermine scientific justifications and the expected management outcomes, especially those involving moose productivity, harvest, and antler trophy quality [7]. We previously observed no clear evidence that predator control increased moose harvest over a 40-year period in south–central Alaska [8]. Nevertheless, reducing predators can interact with density-dependent processes that drive moose population growth, thereby affecting the reproductive potential of moose populations, as well as their age and sex composition—all of which can influence predator–prey dynamics [9]. We provide alternative perspectives on existing management approaches for moose populations subjected to predation in Alaska with the intention of modifying and improving their conservation using the best applicable science. To that end, we organized the body of our review into sections dealing with the policy underpinning the management of large carnivores and the ungulates they prey upon, moose population dynamics, the effects of parasites, diseases, and weather on moose populations, the role of moose density, and the influence of predator control on trophy moose, including tradeoffs between controlling predators and harvesting trophy moose. We conclude that changing paradigms is necessary for moose and predator management, emphasizing how adaptive management can be of value in this process. We integrate conclusions from those sections to provide a broader understanding and perspective on the science-based management of moose and their predators in Alaska.

### Underpinnings of Large Carnivore–Ungulate Policy

The State of Alaska has implemented long-term and often controversial efforts to reduce the abundance of large carnivores, expecting increases in the populations of wild ungulates and, consequently, the human harvests of those animals [8,10,11,12,13]. The State’s management of wildlife is directed by the Alaska Board of Game (BOG), whose seven citizen members are appointed by Alaska’s governor. The BOG receives professional advice from the Division of Wildlife Conservation within Alaska’s Department of Fish and Game (ADFG). The BOG has statutory authority to direct policy pertaining to wildlife, and their harvest and management, sometimes with direction provided by the State legislature, which approves nominations to the BOG [10]. Regulations adopted by the BOG apply to all publicly and privately owned lands in the State, except for National Parks established prior to the Alaska National Interest Land Conservation Act (ANILCA) in 1980, and for Kenai Fjords National Park. BOG regulations do, however, apply to National Preserves, which are adjacent extensions of National Parks, and are also administered by the U.S. National Park Service.

More than 30 years ago, an independent set of hunting regulations and policy guidelines was established by the Federal Subsistence Board (FSB) that apply to all Federally owned lands in Alaska (43CFR, part 51), including the National Parks created or expanded in 1980 by ANILCA, National Preserves, National Wildlife Refuges, the U.S. Forest Service, and the Bureau of Land Management lands. Members of the FSB are charged with assuring that the subsistence needs of “rural subsistence users” are met (this category of users was established in Title VIII of ANILCA). FSB hunting regulations, first enacted in 1992, prioritize the harvest of wild meat for “Federally qualified subsistence users,” which can be defined differently among geographic areas or pertaining to the species harvested (https://www.doi.gov/subsistence/library/history, accessed on 29 January 2026). The FSB can further differentiate subsets of rural users based on historical “customary and traditional uses” of wildlife. In practice, FSB regulations have been similar to those adopted by the BOG; however, this is not a requirement. Legally, the FSB could limit hunting to federally qualified subsistence users on any federal lands in Alaska. Indeed, federal subsistence regulations prioritize the harvest of wild meat for federally qualified subsistence users. Clearly, this strategy compromises the prospective scientific management of ungulate populations exposed to habitat changes or predation by large carnivores.

In 1994, the Alaska legislature passed the Intensive Management Statute (Alaska Statutes §16.05.255 e–g) to codify a management priority for the harvest of meat from moose (*Alces alces*), caribou (*Rangifer tarandus*), and Sitka black-tailed deer (*Odocoileus hemionus sitkensis*); Dall’s sheep (*Ovis dalli*) were added to this priority in 2025. Attempts by the BOG to comply with the Intensive Management (IM) law primarily entail predator reduction programs for large carnivores (grizzly and brown bears, *Ursus arctos*; black bears, *U. americanus*; and gray wolves, *Canis lupus*) across most areas of Alaska in anticipation that those programs will increase the harvests of these ungulates [8,13]. The simplistic intent is to make ungulates killed by predators available, instead, for human harvest. Predator reduction efforts involve both the geographically widespread liberalization of predator hunting regulations [13,14] and the designation of smaller Predation Control Areas, where predators may be killed using aggressive techniques, including aerial gunning by ADFG staff [15,16]. Moose have been a focus for the intensive management of predators across 61% of Alaska’s vast landscape [13], encompassing nearly everywhere that moose occur. The improvement of habitat is also permitted to attain expected levels of ungulate harvests; however, controlled burning and mechanical clearing have not been conducted at a sufficient scale in Alaska to be effective [13]. Moreover, wildfires have a minimal immediate effect on moose harvests [17].

Dual Federal and State hunting regulations render scientific management of wildlife populations difficult and sometimes infeasible. Indeed, the current system mandates that both the State and Federal governments in Alaska give priority to consumptive users of wildlife; State regulations have focused almost exclusively on predator reduction efforts as a means to that end [10]. Under State regulations, which apply to most of Alaska, little consideration is given to the long-term ecological consequences of predator reduction efforts [10,13]. Despite strong opinions, there is general support for prioritizing the use of wild ungulates for meat over trophy or sport hunting [18,19,20,21]. Indeed, a goal of IM is to manage populations so that moose and other ungulates killed by large carnivores are instead available for human harvest. This scheme, however, does not always rest on sound science or lead to the long-term sustainable management of ungulate or predator populations.

## 2. Moose Population Dynamics

**Figure 1:**
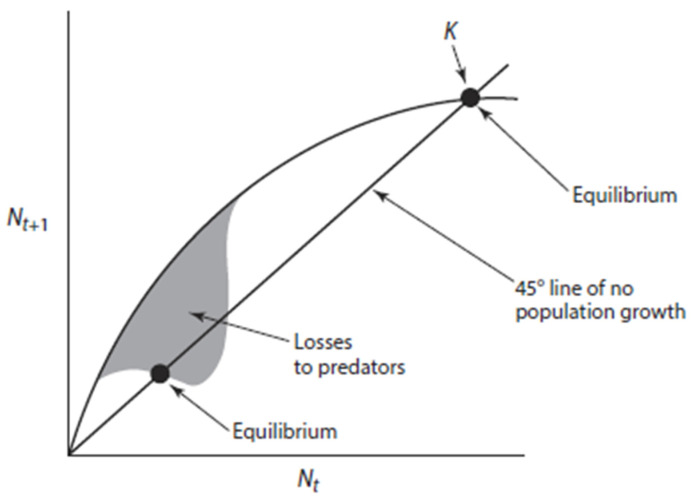
A Ricker-like stock–recruitment curve illustrating the concept of a predator pit where predators can hold an ungulate population at low density. *N_t_* is the ungulates population at one point in time, and *N_t_*_+1_ is the population one reproductive effort later. The shaded area denotes the shape of the recruitment curve from losses to predators. Note the strong points of equilibria and the similar shape of the recruitment curve at *K* (ecological carrying capacity) and at low population size near the bottom of the pit. The equilibrium point at low density makes it difficult for prey to “escape” from the pit (from [42]).

Moose populations in Alaska total between 175,000 and 200,000 animals across an expanse of >1,500,000 km^2^, which includes considerable habitat that remains largely unfragmented [2,13]. Predation on moose, especially neonates, by black and grizzly bears, and on all age classes by wolves, strongly influences the dynamics of moose populations, and in some instances holds populations at low densities relative to the carrying capacity (*K*) of the landscape [22,23,24,25,26,27,28,29,30,31]. Such low-density populations of moose may be in “predator pits” wherein mortality from predation is overwhelmingly additive (sensu [32]) and siphons off any potential population growth despite abundant food resources. This outcome can create a conceptual low-density equilibrium, analogous to that near *K*, which makes it difficult for moose to “escape” from the effects of predation without some level of management intervention (Figure 1).

Controversy exists, however, over whether predation can limit or regulate (sensu [33]) moose populations and the circumstances under which it might do so, or how to determine if those circumstances exist [1,34,35]. Predator pits are just one among many models explaining the predator–prey dynamics of ungulates and large carnivores [31,34,35,36,37,38,39]. Nonetheless, the concept that moose populations are often limited well below their potential population density by predation and can be increased by reducing the number of predators is a fundamental underpinning of predator management in Alaska [13,27,40]. Indeed, managing the level of predation is a key tool used by managers to achieve IM objectives in areas where predation is thought to limit the size of ungulate populations [41].

Density-dependent mechanisms affect the population dynamics of large mammals dramatically ([3,6,7,43,44,45] and many others). Indeed, empirical and experimental evidence strongly support the role of density dependence in regulating populations of cervids [46,47,48,49,50,51,52,53,54], which possess a suite of life history characteristics similar to those of moose, even in the presence of predation [9]. Moose likewise exhibit strong density dependence [55,56,57,58,59,60,61,62,63]. Nevertheless, density dependence was thought to be lacking in some populations of northern cervids, especially at low densities [26,64,65]. Such misperceptions likely resulted from difficulties in understanding the complex interactions of weather with population density, failures to examine populations over sufficient time or densities relative to *K*, time-lags associated with the recovery of nutritional and somatic resources, an intergenerational delay in density-dependent responses, and mistaking an overharvest for a lack of density dependence [9,57,62,63,64,65,66,67].

**Figure 2:**
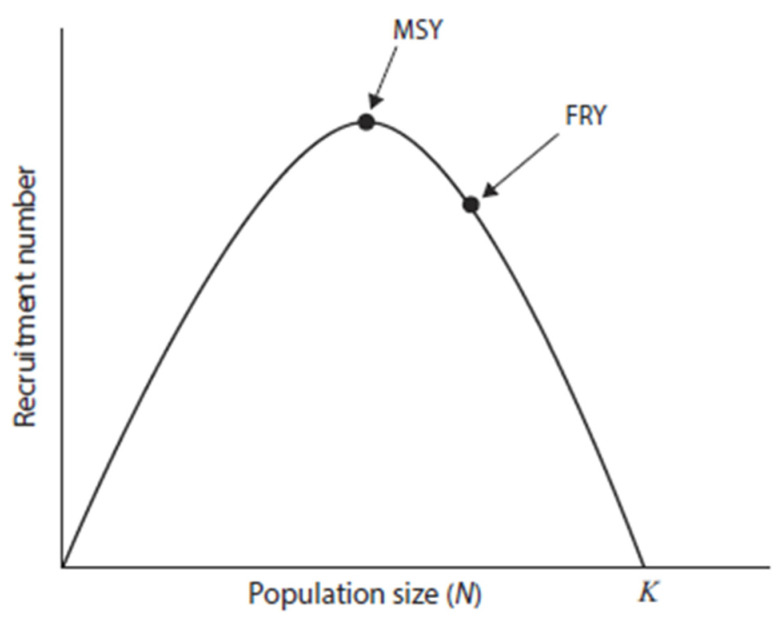
The parabolic relationship between recruitment number (i.e., the number of young successfully added to the population) and population size (*N*) for an ungulate population exhibiting density dependence. MSY is the maximum sustained yield, which is the maximum harvest (or other mortality) that the population can sustain without reducing it, and FRY is the fixed removal yield, a more conservative harvest designed to prevent an incidental overharvest of the population near MSY. *K*, the ecological carrying capacity, is the number of individuals that the environment can support under equilibrium conditions (adapted from [6,7,42]).

Population density, and the accompanying intraspecific competition by moose, which is affected by the proximity of the population to *K*, is primarily mediated by the influence of nutrition on reproduction and the successful recruitment of the young into the population [22,68,69,70,71,72]. Indeed, intraspecific competition for resources is the primary mechanism influencing nutrition and subsequent reproduction in moose and other ungulates ([7] for review); predation risk, however, may also modify resource use by ungulates [73,74,75,76,77,78]. As populations grow from low density toward *K*, intraspecific competition for resources increases, and the per capita availability and quality of food decline; this negatively affects bodily condition and the subsequent successful recruitment of the young into the population (i.e., the recruitment curve in Figure 2). This effect is most pronounced in populations from maximum sustained yield (MSY) to *K* ([6], Figure 2). Indeed, malnutrition can be a leading cause of natural mortality in moose populations near *K* [79]. The young recruited, illustrated in Figure 2, may serve as replacements for harvested adults, a classic outcome for density-dependent species [7,80].

Populations become increasingly food-limited when they exceed MSY and approach or surpass *K* (Figure 2). As a consequence, heightened nutrition following reductions in population size can increase survival and fecundity, thereby compensating for the animals that were harvested [6,45]. Density-dependent responses to reduced population size, especially at or below MSY, include the increased survival of young [45,54,81], larger body mass of neonates [29], and enhanced rates of population growth [4,82,83,84]. In addition, increased litter size [29,83], rapid growth and attainment of large body size [67], high rates of pregnancy [53], and an early age at first reproduction [4,54,84] are associated with a high nutritional plane typical of a population well below *K* [4,6,7,45]. Those density-dependent responses facilitate resilience to harvest and promote the persistence of hunted populations of ungulates [65,80].

Adult females play a pivotal role in the population dynamics of sexually dimorphic ungulates, whereas males are less influential [4,5,82]. This outcome primarily occurs because the sexes of these large, dimorphic mammals sexually segregate from one another for much of the year ([42,85] for reviews). Indeed, sexual segregation is prevalent among Alaskan moose (*A. a. gigas*) [86,87,88,89,90,91]. This differential use of space and other resources by the sexes is related to their morphological and physiological dissimilarities, which affect their forage and habitat selection as well as their predation risk [76,92,93,94,95]. Such differences in the use of space and other resources result in females competing more intensely with other females and their accompanying young than with adult males, especially as the population approaches or exceeds *K* [96]. Indeed, the *X*-axis in Figure 2 should be interpreted explicitly as the adult female component of the population for most sexually dimorphic ungulates [4]—the density of females is responsible for the previously discussed changes in nutrition affecting demographic characteristics [6].

The harvest of males has a comparatively small effect on the population productivity of sexually dimorphic ungulates compared with the harvest of females [4,52,97,98]. Although some after effects may occur from harvesting males [99], those outcomes are comparatively minor compared with those resulting from the harvest of females [6,82]. Harvesting males to sufficiently low numbers that fertilization of females does not occur is unusual [98,100,101], mostly because of the highly polygynous mating systems of sexually dimorphic ungulates, including moose [102,103,104]. Nonetheless, a sufficiently heavy harvest of males can affect their age structure, resulting in a population with younger males [59,105,106] that have smaller body and antler sizes than older males [106,107]. Furthermore, the moose harvested in Alaska are mostly males, with few permits typically issued for females, although rare exceptions have occurred [40,100,108]. In effect, a productive population at MSY with large-antlered males cannot be achieved by a harvest of mostly males, particularly if the population size is between MSY and *K* (Figure 2). Consequently, sources of mortality other than the harvest of males must operate to hold moose at lower densities. Those other sources of mortality include diseases and parasites, severe weather, and predation.

## 3. Parasites, Diseases, and Weather

Moose are beset by a variety of diseases, parasites, and pests, of which only a few are thought to have the potential to affect their populations in North America ([1,109] for reviews). Although chronic wasting disease occurs in other free-ranging cervids in North America, no evidence exists that moose in Alaska harbor that disease [110,111]. Likewise, winter ticks (*Dermacentor albipictus*), which can cause substantial mortality of moose at lower latitudes, have not been reported in Alaska [112,113], although a warming climate might facilitate the spread of parasites northward in the future. Meningeal worms (*Paralaphostrongylus tenuis*) and arterial worms (*Elaeophora schneideri*) can cause debilitating disorders in moose; the requisite alternate definitive and intermediate hosts, however, are absent from Alaska [114,115]. Hydatid disease (*Echinococcus granulosus*), however, occurs in moose in Alaska and may make them more vulnerable to predation by wolves, potentially influencing the population dynamics of those large cervids [116,117]. Evidence that most of these parasites or diseases are causing moose populations to occur at low densities in Alaska is lacking, and the potential effects involving hydatid disease on moose are dependent upon predation by wolves. Consequently, low-density populations of moose are likely the result of factors other than just parasites and diseases.

Abiotic factors also may adversely affect moose populations. Severe weather, especially involving deep snow, has such a potential [1,25,27,118,119,120,121]. The nutritional condition of ungulates can exacerbate the harmful effects of weather when populations are at a high density in relation to *K* and can diminish those effects when populations are at low densities [6,7,53,122,123]. Nonetheless, there are winters of sufficient severity to kill without regard to the physical condition of animals; such winters must, however, be infrequent, or few animals would persist in such environments [42]. Moreover, populations reduced by severe winters may recover quickly because surviving individuals experience reduced intraspecific competition, thereby improving body condition and enhancing reproduction [124].

## 4. Moose Density, Predator Control, and Trophy Moose

The logical conclusion from the foregoing discussion is that predation by large carnivores helps maintain some moose populations at low densities across Alaska. Furthermore, moose populations regulated by predation at levels near or below MSY are in much better nutritional condition than those occurring at higher densities and especially than populations at or near *K*—outcomes that hold importance for both the body and antler size of moose [6].

**Figure 3:**
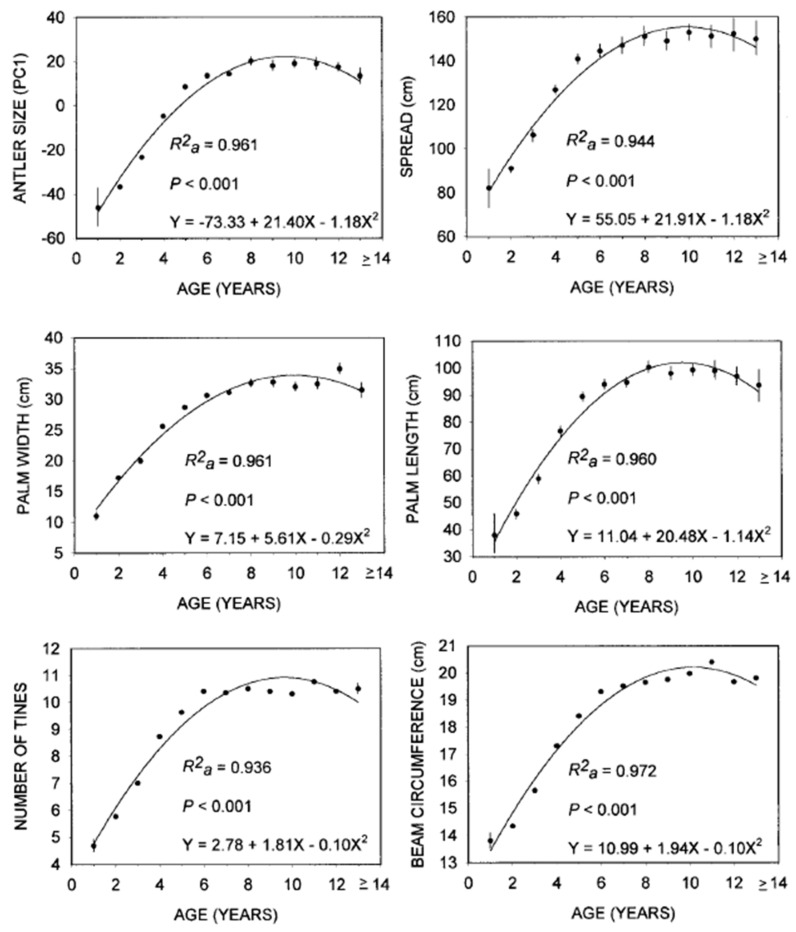
Weighted regression analyses of characteristics related to antler size of Alaskan moose against age. Vertical lines are *SE* (original data [136]; from [135]).

The growth and size of antlers are directly related to age, nutrition, and genetics [106,125,126,127,128,129,130,131,132,133,134]. The growth and shape of antlers in Alaskan moose changes rapidly from 1 to 6 years old, attains a plateau in size for prime-age individuals of 7 to 11 years old, and then regresses somewhat in senescent males aged 12 to 17 years old; this pattern holds for the antler spread, the palm width and length, the number of antler tines, and the beam circumference ([135]; Figure 3).

**Figure 4:**
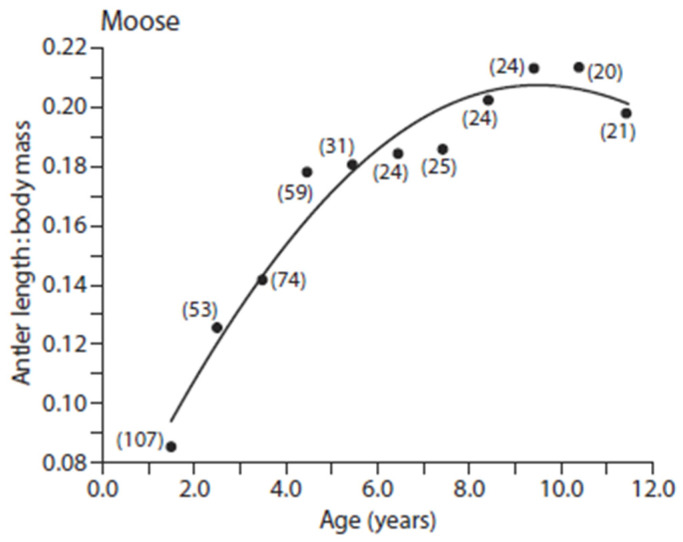
Regression analyses weighted by sample size (in parentheses) of the ratio of antler length (cm) to body mass (kg) against age for Eurasian moose (data from [139]). Regression model: *Y* = 0.04 + 0.83*X* − 0.002*X*^2^, *R_a_*^2^ = 0.967, *p* < 0.0001, and *n* = 11. Note that moose do not fully invest in antlers until adult body mass is achieved (from [42], originally published in [138]; data from [141]).

Male moose typically do not achieve maximum antler size until their body growth is completed at 6–8 years of age [137,138]. As a consequence, young moose do not acquire fully developed antlers at the expense of body growth, likely because of the importance of attaining a large body size, which is critical in male–male combat for mates ([104,138,139,140]; Figure 4).

Connolly [142] argued that the harvest of cervids at MSY (Figure 2) caused population-level shifts from older to younger age classes. Consequently, males would not survive long enough to obtain the resources sufficient to grow trophy antlers under circumstances where populations were near or below MSY. Conversely, McCullough [140] proposed that managing a population at MSY results in a higher proportion of trophy animals. Empirical evidence indicates that harvesting at or near MSY produces deer (*Odocoileus* spp.) with the largest antlers [107,140,143]. This outcome occurs, in part, because females in better nutritional condition produce male offspring that are larger, and remain so over much of their life because of maternal or cohort effects; those males also grow larger antlers [67,71,143,144,145].

Antler size is heritable, providing a reliable indicator of male phenotypic quality [146,147,148]. Hence, strong selection via a selective harvest of large males over a sufficiently long time period holds the potential to affect the genetics that determine the size of their antlers and other horn-like structures [149,150]. Whether strong and persistent selection against the largest (i.e., trophy) males is extensive enough to cause widespread declines in the size of horns or antlers among hunted species of ungulates is debatable, in part because such levels of management are highly restricted and localized [151]. Herein, we use the term “trophy” to mean large-antlered male moose, as indexed by antler spread ([152]; Figure 3). Even when computer simulations of genetic modifications in antlers resulting from a strong selective harvest were generated, moose required many years to manifest such changes [153]. Indeed, the harvest of trophy Alaskan moose over time has not reduced the size of their antlers [106]. We suspect that many hunters would consider a moose with large antlers a trophy even if the antlers were not sufficiently large to qualify for entry into a record book, such as that maintained by the Boone and Crockett Club [106,124,152,154].

Clear patterns in the size of antlers from Alaskan moose within geographic regions and in habitats they occupied were evident when statistically controlled for age [155]. The largest-antlered moose inhabited the Alaska Peninsula and Copper River Delta. River deltas provide extremely productive habitats for moose [156]. Some areas of tundra are not productive habitats for moose, but willows (*Salix* spp.) supply high-quality forage along braided rivers and associated riparian zones in tundra [157]. The productivity of the boreal forest, which dominates the taiga, and where antlers are comparatively small, is dependent largely upon fires that occur sporadically, and it can take many years for suitable forages to regenerate, especially the trees and shrubs that are required by moose during winter [72,75,158]. Finally, moose with small antlers inhabiting southeast Alaska differ genetically from other Alaskan moose [159]. The relationship between antler size and habitat quality, however, is also modified by the population densities of moose relative to *K* [155]. Even excellent habitat can fail to adequately provision populations at high density relative to *K*, resulting in low pregnancy rates and poor survival rates of young [160].

**Figure 5:**
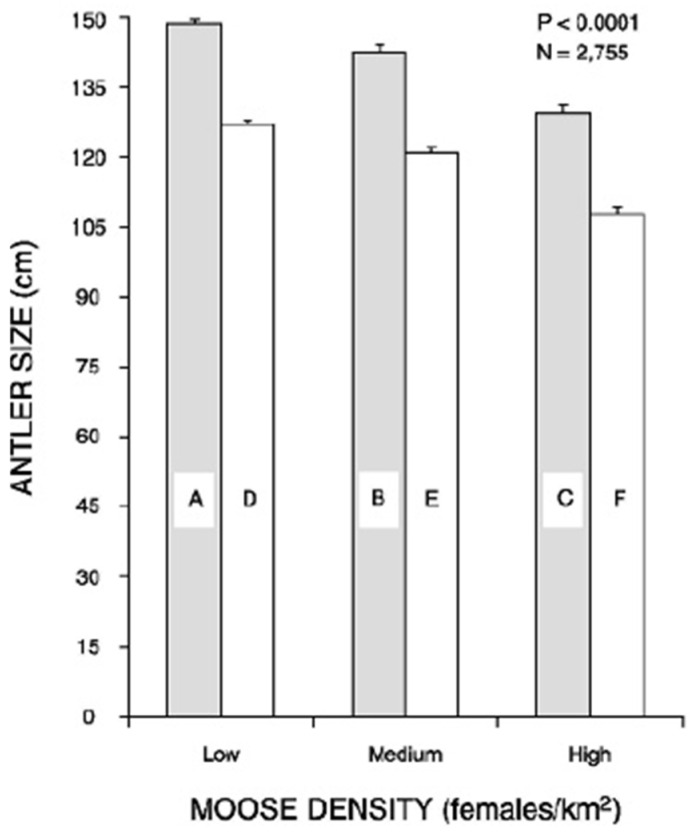
Mean antler size (spread) of Alaskan moose harvested by guided (shaded bars) and non-guided (open bars) hunters from areas of low (<0.21/km^2^), medium (0.21–0.406/km^2^), and high density (>0.406 km^2^) of female moose in interior Alaska, USA. Differing letters on bars indicate significant differences (*p* < 0.05). The *p*-value on the figure represents the overall significance of the comparison of mean antler sizes (from [161]).

Further evidence that habitat influences antler size in moose was provided by Schmidt et al. [161], who reported a strong relationship between the habitat type and antler spread of harvested moose in interior Alaska; moose living in more open areas had larger antlers compared with those from closed boreal forests. Alaskan moose tend to occur in much larger groups than other subspecies [74,104,162] and are harem breeders; other subspecies of moose exhibit a tending bond mating system [103,104]. Some sexual selection for larger antlers in more open habitats may have occurred because of the increased polygyny associated with harem mating and larger group sizes in these animals [104]. The effects of population density on antler size, however, cannot be ruled out. Schmidt et al. [161] documented that moose harvested from low-density populations had larger antlers than those from moderate- or high-density populations, likely because of the negative effects of population density on nutrition. The presence of a professional guide during hunts also resulted in the harvest of moose with larger antlers compared with non-guided hunts; guides often hunted in areas with low moose density (Figure 5).

Under circumstances where predators hold moose at low density in Alaska ([27]; Figure 1), a tradeoff may exist between controlling predators and harvesting trophy moose. Most trophy moose are produced in populations occurring at a comparatively low density [161] that likely are below MSY. Effective predator control may allow moose populations to increase beyond MSY, which results in a reduction in their nutritional condition and, subsequently, their smaller body and antler size, especially if females are not harvested. The occurrence of smaller-antlered moose from areas with high densities [161] indicates that not all moose populations are in predator pits. Moreover, in high-density moose populations that have exceeded MSY because of predator control, a predominantly male-only harvest would serve to lower the age structure of those populations, resulting in a population characterized by younger male moose with concomitantly smaller antlers. Conversely, predators holding moose populations at low density result in moose acquiring large antlers. Consequently, predation is the likely cause of large-antlered moose where those cervids occur at low densities.

Based on an examination of Moose Management and Harvest Reports (Moose Management, Alaska Department of Fish and Game, https://www.adfg.alaska.gov/index.cfm?adfg=moose.management, accessed on 29 January 2026), the primary goal for moose management in Alaska is to increase harvest to obtain meat for human consumption wherever there is an unmet demand, rather than harvesting moose with trophy antlers. What is poorly recognized is that the management of moose for a fixed removal yield (FRY, Figure 2)—a more conservative harvest designed to prevent an incidental overharvest of the population near MSY—also increases recruitment ([4,108]; Figure 2). Although a harvest at FRY is favored to maximize the number of moose taken without overharvesting, a small range of values around FRY may be most practical for management purposes [27,108]. Importantly, a harvest near FRY also promotes large-antlered moose via its effect on nutritional condition—the two objectives of large antlers and a high harvest for meat are not mutually exclusive, so long as females are harvested to achieve that goal.

**Figure 6:**
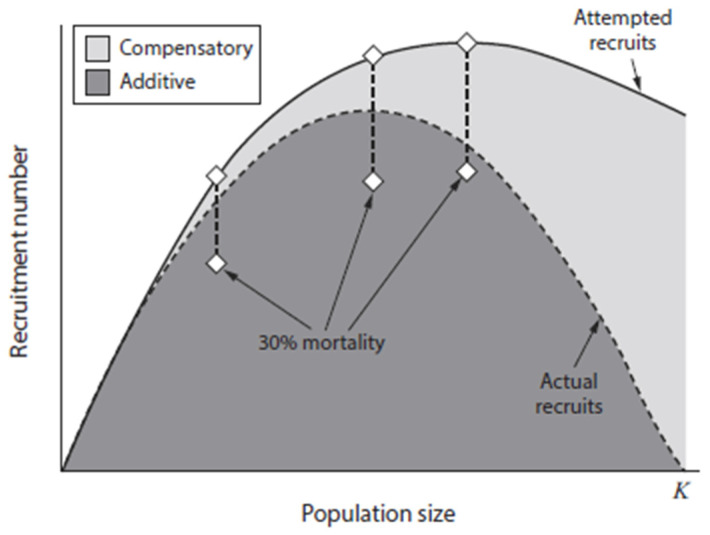
Changes in the number of actual recruits as well as unsuccessful attempts to recruit in relation to the increasing size of an ungulate population. Females attempt to add more young to the population than can be sustained by the environment as a function of carrying capacity (*K*). Note that mortality becomes increasingly more compensatory (one source of mortality substitutes for another) as the population approaches *K*. In contrast, a similar level of mortality (30%) becomes increasingly additive (one source of mortality is added to another) as population size backs further away from *K*, because the number of young that females attempt to recruit approaches the number of young they can recruit given improvements in nutrition (adapted from [54] from [42]).

Predator control that releases moose from low densities (Figure 1) can potentially result in an increasing moose population [27]. Nevertheless, a harvest of primarily male moose will do little to prevent that population from reaching and then exceeding MSY, leading to a lower plane of nutrition as the population approaches *K*—circumstances that result in more moose with smaller antlers. Because predominantly males are harvested, this management approach has limited effects on the overall population dynamics of moose. Moreover, as populations approach *K*, killing predators will have progressively fewer influences on moose populations because mortality becomes increasingly compensatory (sensu [32]) and predator control becomes largely inconsequential, because those moose not killed by predators would die from other causes, such as severe weather ([6,54]; Figure 6). Wise management, then, requires that the relationship of the moose population to *K* be known before initiating or terminating predator control. Not having this knowledge may also render male-only harvests for populations at or beyond MSY less advantageous, making harvests designed to acquire more meat or large-antlered moose unattainable. Additionally, too little attention has been paid to the status of predator populations that are controlled to promote moose harvests, although several comprehensive monographs dealing specifically with large carnivores in Alaska are available [163,164], among others. Unlike some populations at lower latitudes, grizzly bears and gray wolves in Alaska are not listed as threatened or endangered by the U.S. Fish and Wildlife Service (https://ecos.fws.gov/; accessed on 29 January 2026). Nevertheless, local populations of large carnivores require monitoring to ensure their sustainability [165]. Importantly, we caution against using unreliable predator–prey ratios to assess the status of bears and wolves in relation to moose populations [9,166,167].

## 5. Changing Paradigms for Moose and Predator Management

A 40-year analysis in south-central Alaska demonstrated that the current approach of reducing wolves and bears did not result in increased moose harvests, thereby failing to provide a useful index to the effects of killing predators on the harvests of those large ungulates [8]. Numerous variables influence the successful harvesting of moose [168], which clearly responds to factors in addition to predator removal. Moreover, an increasing population of large carnivores will not necessarily reduce the hunter harvest of ungulates [169]. Since the passage of the IM law in 1994, no credible documentation exists that demonstrates that harvests of moose have increased in areas with predator reduction efforts, whether from the liberalization of predator hunting regulations or more aggressive efforts, such as shooting predators from helicopters by ADFG staff in designated predator control areas. Indeed, most studies concluding that predator control is effective in promoting increases in ungulate populations contained experimental biases, such as a lack of controls, the absence of randomization, low replication, a short temporal length, and confounding variables, leading to a lack of credible scientific evidence that predator removal was an effective long-term management strategy [10,170], despite claims to the contrary [171].

The present-day strategy of harvesting predominantly males will not provide moose harvests near MSY, as this cannot be achieved without harvesting females—a conclusion also reached by Boertje et al. [40,172,173]. Indeed, only 3–4% of moose in Alaska are harvested annually [174]. Moose have a maximum intrinsic rate of increase of *r_max_* = 0.35–0.44 [59], which would require well over a ten-fold higher harvest to bring about low-density populations. Increased effort, however, will be necessary to convince the public that harvesting females is a viable management strategy [175]. Importantly, a tradeoff exists between controlling predators and harvesting trophy moose. Where predator control results in moose populations increasing beyond MSY, the size of antlers would decline, thereby constraining the ability of some guides and their clients to harvest trophies. This same outcome would occur for self-guided hunts, where the size of moose antlers also would decline. Conversely, harvests at FRY would result in both more meat and larger antlered moose.

A focus only on the increased killing of predators to enhance moose harvests is a poor paradigm for moose management [8]. Indeed, the most pressing yet challenging need for biological information concerning the wise management of moose and their predators is knowing the density of moose and the proximity of populations to *K* [6,7,8]. Moose in populations near *K* tend to be in poor physical condition, where predator control will do little to provide more moose for harvest because most mortality is compensatory (Figure 6). As the population increases in size beyond MSY, the importance of predation becomes less influential because progressively more mortality is compensatory. Moose populations at a low density may benefit from the killing of predators, provided they are in a predator pit (Figure 1), when most mortality at low density is additive (Figure 6). Caution should be taken, however, to employ methods that ensure moose are not limited by poor habitat rather than by predation. Moreover, harvesting female moose in populations below MSY will not increase productivity but will reduce population size because the recruitment of young does not compensate for removals by harvest (Figure 2). Patently, differences in the density of moose among populations or changes in density within a population may fail to provide a reliable index of compensatory or additive mortality, because *K* can vary across areas or time. Furthermore, animal density per se should not be used as an index to habitat quality [176]. Hence, additional data are necessary to ascertain the proximity of the population to *K* [6,7,172,173,177]. The ADFG has considerable expertise in reliably estimating moose populations from aerial surveys [178]; moreover, surveys can be modified to incorporate the effects of habitat [158]. Aerial surveys are expensive [179,180], and such surveys may not be a yearly necessity. An estimate of female density alone, however, is not sufficient for the wise management of moose, especially in environments with a full complement of natural predators. Measures of the nutritional condition of moose and other population characteristics can be used to help estimate the relationship of the moose population to *K* [6,177,181]. A suite of variables is available to assist with such an evaluation, provided that care is taken to employ multiple approaches ([6]; Table 1). Obtaining data from the female component of the population is essential to evaluate population dynamics of moose and other ungulates. Gathering information on nutritional condition and associated population characteristics (Table 1) is superior to assessing forage availability and range condition because of time lags in the response of vegetation to perturbations; the effects of variable weather can also obscure the interpretation of results [6,122,124]. Indeed, forage-based methods for estimating *K* and determining the nutritional or reproductive status of moose are available [173,182,183], but are labor-intensive; forage measurements may also lag behind declines in ungulate populations [7]. Moreover, browsing intensity by moose was not predictive of calving success [184], and willow quality can vary markedly in response to annual weather patterns [28]. Foraging in seasons other than winter also plays an important role in the nutrition of moose [72], necessitating a year-round assessment of vegetation abundance and quality. Moose would be expected to respond reproductively to such variation in forage quality, mostly when populations were near *K* and forage was limited. Furthermore, moose sexually segregate in spring and winter [86,87,89], necessitating that ranges used principally by females be assessed, an important factor that is frequently overlooked [42].

When sufficient data for understanding moose population dynamics are unavailable because of limited time or expense, we propose implementing an adaptive management approach [10,185,186], which is also advocated in Alaska’s Intensive Management Protocol, but has yet to be undertaken effectively [41]. This procedure could vary the harvest of females (and males) and monitor selected nutritional variables and population characteristics (Table 1) to help wildlife managers determine whether the harvest is congruent with desired goals. In low-density populations, similar data could be obtained by capturing, sampling, and collaring females rather than relying on harvested moose as sources of information to meet those goals. We maintain that implementing an approach that combines the density of female moose with measures of nutritional condition and population characteristics that change across population density offers the best opportunity for prudent management.

For example, predator-regulated populations would exhibit low numbers (or densities) relative to *K*, adult females in good physical condition, low recruitment despite high birth rates, few pauses in annual reproduction, high twinning rates, mortality of the young primarily resulting from predation, additive mortality, a higher proportion of trophy-sized males, and more limited effects from severe winter conditions. Management recommendations for achieving a maximum sustained harvest (or harvest at FRY [Figure 2]) with more trophy males include curtailing the harvest of females, antler size restriction when harvesting smaller males, and reducing the number of predators. Outcomes from such management would be assessed and readjusted annually, if necessary, to increase the female component of population size and move the harvest toward FRY.

Habitat-limited populations would comprise high numbers (or densities) of moose relative to *K*, exhibit adult females of poor condition, have low birth and recruitment rates, have low twinning rates, have pauses in annual reproduction, the mortality of young would be the result of disease, malnutrition, and predation (i.e., compensatory mortality), and there would be fewer trophy-sized males and pronounced effects from winter severity. Management recommendations for attaining a maximum sustained harvest with more trophy males include a substantial harvest of females and an initial antler size restriction when harvesting smaller males. Predators need not be harvested extensively because of compensatory mortality. Annual assessment and adjustments would be undertaken with the objective of moving the population toward FRY.

Populations at or near FRY would exhibit characteristics similar to, but slightly less pronounced than those of predator-regulated populations, except that they would occur at moderate numbers (or densities) relative to *K*, and sustain a much larger harvest of adult females and trophy males. Annual assessments, then, would allow for the evaluation of variation in life history and population characteristics (Table 1) to maintain the population near FRY. A moderate kill of predators from sport hunting should be considered. The number of adult females harvested, their physical condition, and the antler size of males, however, are variables of primary interest.

We caution, however, that nonlinearities in the variables in Table 1, as well as changes in recruitment number with population size (Figure 2), often complicate the interpretation needed for adaptive management. Maternal and cohort effects, which can delay effects of density dependence, are widespread among cervids [67,143,144], including moose [71], and might further confuse interpretations, as can time lags in density-dependent responses to manipulations [187]. Despite potential difficulties, the framework outlined herein (and the wide range of characteristics in Table 1) offers a robust scientific approach to support the management of Alaska’s moose populations. We provide a more formal approach to addressing adaptive management in Supplementary File S1.

The State hunting regulations in most of Alaska are currently designed to incentivize hunters to take more predators, especially brown bears [3,8,188,189], without adequate assessment of the relationship of ungulate populations to *K*. These regulations include allowing the sale of brown bear skulls and hides, eliminating tag fees, baiting, not closing hunting seasons, and bag limits of two bears per year [14]. The sale of bear hides and skulls is the commercialization of wildlife, which has been widely criticized [190]. Moreover, this practice is not in line with the tenets of the North American Model of Wildlife Conservation, a cornerstone of wise management [190]. We suggest that many controversies concerning predator control derive largely from the implementation of attempts to satisfy the Intensive Management protocols for Alaska’s ungulates and their predators. More science and public education and less politics would promote a management approach that culminates in the harvest of more moose with larger antlers, while moderating predator control where it is ineffective in achieving management objectives.

Other aspects of predator control are neither adequately accounted for by those responsible for managing ungulates and their carnivores in Alaska, nor considered in the Intensive Management protocol. For instance, tourism is a critical component of Alaska’s economy [191]. Indeed, hunters contribute to the economy by purchasing licenses and tags, various types of transportation, food, and equipment, and hiring professional guides [10,191,192]. Although guides likely will continue to harvest trophy moose under the current management regime, the intensive predator control measures across large areas of the State [13] will make that task more difficult and lead to dissatisfaction among some non-resident and resident hunters, particularly where predator control results in small-antlered moose. Moreover, such aggressive levels of predator management are unacceptable to many members of the public [193]. Those outcomes hold importance for Alaska’s professional guides and for tourism (both non-resident hunters and others wishing to view or photograph large mammals), and thereby the overall economy of the State. These economic factors are not yet a primary consideration in predator management in Alaska. Indeed, the National Research Council [10] concluded that Alaska’s predator control programs did not effectively assess the full economic costs and benefits of those management activities, a continuing difficulty. Clearly, an amalgamation of a modern understanding of population ecology with pertinent social factors and public education will be necessary to resolve ongoing issues concerning the wise management of moose and the large carnivores that prey upon them.

## 6. Conclusions

A convoluted mix of State and Federal regulations complicates the management of moose, other ungulates, and large carnivores in Alaska. The State’s Intensive Management policy, which allows the Board of Game to increase the harvest of moose and other ungulates for meat wherever an unfulfilled demand occurs, is especially problematic. This management protocol does not necessarily incorporate a comprehensive understanding of predator–prey dynamics in decision making. Intensive predator control has been undertaken, sometimes without adequate information concerning the nutritional condition and population size of moose in relation to *K* or the status of predator populations, which clearly does not encompass the best science. In addition, the killing of predators throughout much of Alaska is the result of the liberalization of regulations without sufficient data on the outcomes of such programs. Although predators clearly hold the potential to regulate moose numbers, studies suggesting that the effects of predator removals were effective in promoting increases in ungulate populations are fraught with experimental biases—a rethinking of predator control, including the types and scope of research necessary to understand its influences on ungulate populations, is overdue.

Modern concepts involving the population ecology of moose, especially those related to density dependence, need to be incorporated into management decisions, including the concepts that a maximum harvest of meat cannot be attained without harvesting females, and that such a harvest can increase the nutritional condition of moose, the recruitment of young, and the number of trophy-antlered males. Management would benefit from additional, carefully designed studies to assess the effects of predator removal on moose population dynamics, including data on the status of predator populations. If data are insufficient or unavailable because of limited time or expense, we recommend employing an adaptive management approach, wherein objectives are repeatedly modified to examine expected density-dependent responses to changes in harvest and predator removals. We also advocate that the social aspects related to predator control and moose harvest play a larger role in management policy, particularly educating the public about the population dynamics of large mammals, and providing more consideration of the economic effects of predator control on tourism in Alaska’s economy.

## Figures and Tables

**Table 1:** Variation in life history and population characteristics of ungulates in relation to the proximity of the population to MSY (maximum sustained yield) and *K* (ecological carrying capacity) (modified from [6,7]).

Life History and Population Characteristics	≤MSY	Near *K*
Physical condition of adult females	Better	Poorer
Pregnancy rate of adult females	Higher	Lower
Pause in annual reproduction by adult females	Less likely	More likely
Yearlings pregnant ^a^	Usually	Seldom
Corpora lutea counts of adult females ^a^	Higher	Lower
Litter size ^a^	Higher	Lower
Age at first reproduction for females	Younger	Older
Weight of neonates	Heavier	Lighter
Mortality of young	Additive	Compensatory
Diet quality	Higher	Lower
Population age structure	Younger	Older
Age at extensive tooth wear	Older	Younger
Forage quality	Higher	Lower

^a^ Some species of ungulates may exhibit limited variability in particular characteristics.

## Data Availability

No new data were created or analyzed in this study. Data sharing is not applicable to this article.

## Supplementary Materials

The following supporting information can be downloaded at https://www.mdpi.com/article/10.3390/ani16030472/s1. File S1: Developing an Adaptive Management Model; Table S1. Covariates to be measured derived from population and life history characteristics.
