# Supplementary material for: Tradeoffs Among Predator Control, Moose Harvests, and Trophy Antlers: Principles Pertinent to Managing Alaska’s Wildlife
Source: Animals (Basel). 2026 Feb 3;16(3):472. doi: 10.3390/ani16030472 (PMC12897136; doi:10.3390/ani16030472)
Supplement: Supplementary file 1 [file animals-16-00472-s001.zip › animals-4028919-supplementary.pdf]

File S1, Supplemental Materials:

We propose an adaptive management framework for developing a management model to guide decisions whether moose populations are (A) well below MSY and limited by predation or (B) near K and limited by habitat. We suggest some similar framework should have been part of ADFG's program of predator control research for years. The suitability of predator control depends greatly on the moose population relative to *K*; however, carrying capacity is notoriously difficult to measure in the field. Nonetheless, variables related to animal condition and reproduction can be used as proxies indicating populations well below MSY and those approaching K (Table S1).

The first step is to select a suite of variables that can be measured efficiently in the field to develop a model, predicting the probability of scenario A or B. The model can be expressed as a natural log function of the form: $\ln(\text{pr } Y) = X_0 + B_1 (X_1) + B_2(X_2) + \ldots.. B_i(X_i)$ because probability is bounded by 0 and 1 and log functions transform data to be relatable within that context.We suggest populating the model with covariates derived from the population and life history traits described in Table S1.

Table S1. Covariates to be measured derived from population and life history characteristics (Table 1)

Characteristic	Measured Covariate
Physical condition of adult females	% females with poor condition index
Litter size	# young per female in June
Mortality of young (1)	% of young removed by predation during summer
Mortality of young (2)	% of young dying in all seasons from causes unrelated to predation
[a]Forage quality (1)	forage abundance by hectare
[a]Forage quality (2)	Digestible energy and protein per kg
[a]Browsing intensity	% current annual growth browsed
Trophy males	% trophy males in the harvest

[a]We caution that forage may be subject to time lags in relation to moose population density.

At least 3 study areas should be selected and data for covariates in Table S1 should be obtained in the field during the first year for each study area. Those data are then applied to the model with probability of scenario A (or B by default) set at a prior probability of 0.5, or essentially uninformative. Model coefficients and their sampling variances are estimated by Monte Carlo sampling of each covariate constrained by its mean and standard deviation, and iterating model computations 1,000 or more times. In addition, moose density or population is estimated. Data from each study area are combined.

During the second year wolves are killed during winter and bears in the following spring at a level likely to reduce them significantly and affect the moose population in some manner. Following predator removal, 2 variables are measured: moose population or density in early autumn, and % young surviving to age 1 year. During the 3rd year, predators are again suppressed as far as possible, and the moose population and % young surviving to year 1 again measured. After each intervention, if the moose population in autumn grows and % young surviving to age 1 increases, we assume a higher outcome probability for the initial model indicating predator limitation. So, after the first year, instead of a probability of 0.5 we give the model outcome a probability of 0.75 and recalculate the coefficients for each covariate. If the population of moose does not grow and the proportion of young surviving to age 1 does not increase, we reduce that probability to 0.25 and recalculate the model. In the third year, we again measure the moose population and the mortality of young. These data should offer up the most definitive results and if the moose population continues to grow, the original model for predation is given a value of 0.95 and the coefficients for each covariate are recalculated for that result. If the population does not respond and the mortality of young does not decline, the next iteration of the original model is given an outcome probability of 0.05, meaning a very low chance that the outcome is true. The original model is then estimated again using that outcome.

Ultimately, we converge on a model with robust coefficients for covariates that can be used to guide management of other areas where the effects of predation are to be examined. Computing the model with data from 3 different study areas should introduce variation owing to local weather and environmental conditions to the model. Predator removal must be effective to influence the moose population. Finally, after 5 years of intense predator suppression and moose population increase, the covariate data used in the first year should be obtained again and applied to predict the probability of scenario A or B. If the model is valid, scenario B should be favored. We stress that predator removal must be sufficient to measurably change those moose population and life history traits. If that cannot be achieved within the designated study areas, it calls into question the entire premise of attempting to suppress predation as a means to increase moose populations under any scenario.
